# *AMT1;1* transgenic rice plants with enhanced NH_4_^+^ permeability show superior growth and higher yield under optimal and suboptimal NH_4_^+^ conditions

**DOI:** 10.1093/jxb/ert458

**Published:** 2014-01-13

**Authors:** Kosala Ranathunge, Ashraf El-kereamy, Satinder Gidda, Yong-Mei Bi, Steven J. Rothstein

**Affiliations:** Department of Molecular and Cellular Biology, University of Guelph, Guelph, ON, Canada, N1G 2W1

**Keywords:** Ammonium transporter, assimilation, gene expression, permeability, rice, transgenic

## Abstract

The major source of nitrogen for rice (*Oryza sativa* L.) is ammonium (NH_4_^+^). The NH_4_^+^ uptake of roots is mainly governed by membrane transporters, with *OsAMT1;1* being a prominent member of the *OsAMT1* gene family that is known to be involved in NH_4_^+^ transport in rice plants. However, little is known about its involvement in NH_4_^+^ uptake in rice roots and subsequent effects on NH_4_^+^ assimilation. This study shows that *OsAMT1;1* is a constitutively expressed, nitrogen-responsive gene, and its protein product is localized in the plasma membrane. Its expression level is under the control of circadian rhythm. Transgenic rice lines (L-2 and L-3) overexpressing the *OsAMT1;1* gene had the same root structure as the wild type (WT). However, they had 2-fold greater NH_4_^+^ permeability than the WT, whereas *OsAMT1;1* gene expression was 20-fold higher than in the WT. Analogous to the expression, transgenic lines had a higher NH_4_^+^ content in the shoots and roots than the WT. Direct NH_4_^+^ fluxes in the xylem showed that the transgenic lines had significantly greater uptake rates than the WT. Higher NH_4_^+^ contents also promoted higher expression levels of genes in the nitrogen assimilation pathway, resulting in greater nitrogen assimilates, chlorophyll, starch, sugars, and grain yield in transgenic lines than in the WT under suboptimal and optimal nitrogen conditions. *OsAMT1;1* also enhanced overall plant growth, especially under suboptimal NH_4_^+^ levels. These results suggest that *OsAMT1;1* has the potential for improving nitrogen use efficiency, plant growth, and grain yield under both suboptimal and optimal nitrogen fertilizer conditions.

## Introduction

Nitrogen is an essential nutrient for plant growth and development and is often the major limiting nutrient for plant productivity (Sonoda *et al.*, 2003). The major source of inorganic nitrogen that is available for paddy rice in submerged soil is the ammonium ion (NH_4_^+^; Tabuchi *et al.*, 2007). Ammonia gas (NH_3_) in water is a weak base that protonates rapidly to form NH_4_^+^ ions in paddy soil (Kleiner, 1981). In addition to its abundance in paddy fields, NH_4_^+^ is the preferred nitrogen source for rice and many other plant species over nitrate due to the lower energy requirement for assimilation by roots (Bloom *et al.*, 1992). However, NH_4_^+^ acquisition and translocation to the shoot can be enhanced by nitrate (Kronzucker *et al.*, 1999).

In the past decades, due to the introduction of high-yielding rice varieties, nitrogen fertilizer (mainly NH_4_^+^) use has increased many fold (Bouwman, 1997). However, the uptake efficiency is known to vary from 30% to 80% (Hoque *et al.*, 2006), with the rest of the nitrogen fertilizer escaping into the environment. Therefore, it is important to find ways to enhance nitrogen use efficiency (NUE) in rice, one of the major food crops in the world. One plausible method could be to increase NH_4_^+^ transport efficiency in roots and shoots and thereby enhance subsequent higher assimilation of nitrogen in plants. Since NH_4_^+^ ions are the preferred nitrogen source for rice, introducing high-affinity NH_4_^+^ transporter genes into plants might lead to an increase in nitrogen uptake in roots and NH_4_^+^ permeability of shoots.

The first NH_4_^+^ transporters to be isolated from any organism were two related high-affinity NH_4_^+^ transporters from yeast (Marini *et al.*, 1994) and *Arabidopsis* (Ninnemann *et al.*, 1994). Related NH_4_^+^ transporter proteins have since been found in several species of plants including rice (von Wiren *et al*., 1997). In rice, ammonium uptake in roots and transport in shoots is mediated by NH_4_^+^ transporters belonging to the *OsAMT* family, which contains 10 *OsAMT*-like genes (Suenaga *et al.*, 2003). Some of the genes in the *OsAMT* family have been partially characterized (Sonoda *et al.*, 2003; Hoque *et al.*, 2006). The *OsAMT1* subfamily consists of three distinct members: *OsAMT1;1*, *OsAMT1;2*, and *OsAMT1;3*. These three members share high sequence similarity to each other and also to other AMT1-type NH_4_^+^ transporters previously identified from other plant species (Sonoda *et al.*, 2003; Suenaga *et al.*, 2003). Since these members exhibit different affinities for NH_4_^+^, it is likely that plants can utilize a wide range of soil NH_4_^+^ concentrations. Specifically, *OsAMT1;1* is known to be a prominent member in this subgroup, showing a constitutive expression pattern in shoots and roots (Sonoda *et al.*, 2003). In the past, it has been suggested that *OsAMT1;1* is localized in the plasma membrane and that its expression (up- or down-regulation) is NH_4_^+^ responsive (Hoque *et al*., 2003; Sonoda *et al.*, 2003). However, proper fluorescence-based localization studies and novel expression studies with quantitative real-time PCR (qRT-PCR) are still necessary to prove this claim.

Overexpression of the *OsAMT1;1* gene in rice was shown to result in the accumulation of higher levels of NH_4_^+^ and impairment of plant growth (Hoque *et al*., 2003). It was hypothesized that excessive NH_4_^+^ inhibited plant growth due to its toxicity. However, detailed physiological studies—such as (i) NH_4_^+^ permeabilities of roots; (ii) subsequent responses in the nitrogen assimilation of shoots; and (iii) yield studies under different NH_4_^+^ fertilizer regimes—are still lacking. In the present study, the previous characterization of the *OsAMT1;1* gene was extended using a multidisciplinary approach including molecular biological, physiological, and biochemical methods. *OsAMT1;1* transgenic lines have a higher NH_4_^+^ permeability in roots that increases NH_4_^+^ uptake, and subsequently increases ammonium assimilation in the shoot under suboptimal, optimal, and high NH_4_^+^ levels in the medium. It also enhances overall plant growth and yield under suboptimal and optimal levels. High levels of NH_4_^+^ lead to toxicity and thus slower growth in wild-type plants, and this effect is even stronger in the overexpression lines. These changes also lead to an increased expression of a number of nitrogen assimilatory genes.

## Materials and methods

### Plant material and growth conditions

Rice seeds (*Oryza sativa* L.), variety Kaybonnet, and transgenic lines overexpressing the putative ammonium transporter gene, *OsAMT1;1* (*LOC_Os04g43070.1*), were used in this study. *OsAMT1;1* was one of the NUE candidate genes identified in a previous study, and its overexpression was driven by a ubiquitin promoter (Bi *et al.*, 2009). Seeds were germinated between moistened paper towels placed in Petri dishes in a climatic chamber (day/night rhythm, 12/12h; 27/22 °C; light intensity, 500 μmol m^–2^ s^–1^). Six days after germination, the seedlings were transferred to an aerated hydroponic system as detailed previously by Miyamoto *et al.* (2001) and Ranathunge *et al.* (2003). Three days later, plants were divided into three groups and transferred to hydroponic cultures supplemented with three different levels of (NH_4_)_2_SO_4_ as the nitrogen source; 30 μM (low), 300 μM (optimum), and 3000 μM (high). During the growth period, aerated nutrient solutions were renewed weekly. The plants were grown in the same environmental conditions as those used for seed germination.

### Plant selection, *OsAMT1;1* expression analysis, and basic phenotypic measurements

The *OsAMT1;1* transgenic rice lines were carrying the *PMI* (*PHOSPHOMANNOSE ISOMERASE*) gene as the selectable marker and were initially screened using standard PMI test strips (Strategic Diagnostics Inc., USA; Miles and Guest, 1984; Negrotto *et al.*, 2000). Antibodies specific to the PMI protein were coupled to a colour reagent and deposited as lines on the strip, allowing for easy and quick detection of positively transformed plants. For PCR-based genotyping, RT-PCR was used to confirm the transgenic plants further (Supplementary Fig. S1 available at *JXB* online). Rice *Actin-2* was used as the control. The primer sequences for *OsAMT1;1*and *PMI* were 5′-TCGTGCTTGGCACCTTCC-3′ (forward) and 5′-TGGTGAACGACCCGGG-3′ (reverse), and 5′-CGCCAGCCTGTTGAATATGC-3′ (forward) and 5′-ACGTTGCATCGCCTTCGAC-3′ (reverse), respectively.

Transgenic plants (L-2, L-3, and L-5), WT, and azygous non-transgenic plants (L-2 neg., L-3 neg., and L-5 neg.) were used for qRT-PCR analysis. For each measurement, three technical and three biological replicates were used. Relative quantity was calculated using the 2^–ΔΔT^ method (Livak and Schmittgen, 2001).

For basic phenotypic and other measurements, T_4_ homozygous seedlings of the two transgenic lines with the highest expression levels of the *OsAMT1;1* gene (L-2 and L-3) were used along with the WT plants. Pre-germinated seedlings were transferred to hydroponics with different levels of NH_4_^+^ (30, 300, and 3000 μM) as the nitrogen source. Fourteen days later, plants were harvested, and shoot height, root length, and plant biomass (dry weight of shoots and roots) were measured. In some experiments, plants were grown for 4 weeks for further root phenotypic studies. Twelve replicates were used for each measurement.

### Expression analysis of the *OsAMT1;1* gene under different NH_4_^+^ dosages

One-month-old, transgenic and WT plants grown in optimum nitrogen (300 μM NH_4_^+^) were transferred to the nutrient solution with different levels of nitrogen (30, 300, or 3000 μM NH_4_^+^). Two hours later, plants were harvested and frozen immediately in liquid nitrogen. Total RNA was extracted from the young leaves and the adventitious roots using TriZol reagent (Invitrogen, Carlsbad, CA, USA) and an RNEasy RNA extraction kit (Qiagen Inc., Toronto, ON, Canada). To eliminate genomic DNA from RNA, the samples were treated with RQ1 RNase-free DNase enzyme (Promega Corporation, Madison, WI, USA). The cDNA was synthesized from total RNA with qScript cDNA SuperMix from Quanta Biosciences (Gaithersburg, MD, USA) and used for qRT-PCR analysis. The PCR primers were designed using the Applied Biosystems (Forster City, CA, USA) software Primer Express 2.0. The qRT-PCR was carried out and analysed with a 7300 real-time PCR system (Applied Biosystems) according to the manufacturer’s instructions. Constitutively expressed *Actin-2* was used as the endogenous control. As described earlier, three technical and three biological replicates were used for each measurement, and relative quantity was calculated using the 2^–ΔΔT^ method (Livak and Schmittgen, 2001).

To analyse the expression levels of the *OsAMT1;1* gene at different times of the day, leaf samples of WT plants were collected at 09:00, 12:00, 18:00, and 22:00h from WT plants grown in optimum nitrogen. Samples were immediately frozen, ground, and used for RNA extraction. Preparation of cDNA and analysis of relative expression levels using qRT-PCR were performed as described earlier.

### Transformation and localization studies

To generate the green fluorescent protein (GFP)::OsAMT1;1 construct, a 1497bp fragment of the *OsAMT1;1*gene was amplified from genomic DNA by AMT1;1HindIII-F (CCCAAGCTTGGGATGGCGACGTGCGCGG) and AMT1; 1EcoRI-R (CCGGAATTCTTACACTTGGTTGTTGCTGTTG GAGGCA) primers. The amplified fragment of *OsAMT1;1* was cloned into the molecular cloning site of the pRTL2dNS/mGFP-MCS vector (Shockey *et al.*, 2006; kindly provided by Dr Robert T. Mullen, University of Guelph, Canada) between the *Hin*dIII and *Eco*RI restriction sites to drive GFP expression. The resulting construct was transformed into *Escherichia coli*, and midi prep plasmid isolation was done according to the manufacturer’s instructions (Qiagen Inc.). Onion (*Allium cepa*) epidermal cells were prepared for biolistic bombardment as described previously (Scott *et al.*, 1999). Transient transformations were performed using 5 μg of the plasmid DNA with a biolistic particle delivery system (Bio-Rad Laboratories Ltd, Mississauga, Canada) as described previously by Shockey *et al.* (2006). To confirm successful transformation further, a plasma membrane-localized red fluorescent protein (RFP) pRTL2-RFP RAC3 marker (Shockey *et al.*, 2006; provided by Dr Robert T. Mullen) was used together with the GFP::OsAMT1;1 vector for biolistic bombardment. Following bombardment, cells were incubated for 36–40h to allow for expression and sorting of the introduced gene product(s) and then processed for fluorescence microscopy. Epifluorescent images of onion epidermal cells were acquired using a Zeiss Axioskop 2 MOT epifluorescence microscope (Carl Zeiss, Germany).

### Expression analysis of genes involved in the nitrogen assimilation pathway

To analyse the expression levels of genes involved in the nitrogen assimilation pathway, such as cytosolic glutamine synthetase (*GS1.1*), chloroplastic glutamine synthetase (*GS2.1*), Fd-glutamate synthase (*Fd-GOGAT*), and glutamate dehydrogenase (*GDH*) in shoots as well as *GS1.2*, *GS2.1*, *NADH-GOGAT*, and *GDH* in roots, plants were transferred to a hydroponic system with different levels of nitrogen (30, 300, or 3000 μM NH_4_^+^). Two hours later, shoot and root samples were collected and immediately frozen in liquid nitrogen. Frozen samples were then ground and used for RNA extraction. Preparation of cDNA and analysis of relative expression levels using qRT-PCR were performed as described earlier. The sequences of forward and reverse primers for the above genes are shown in Supplementary Table S1 available at *JXB* online. *Actin-2* was used as endogenous control.

### Root structural study

Healthy adventitious roots of 1-month-old, transgenic and WT plants grown in optimum nitrogen (300 μM NH_4_^+^) were selected for the structural study. Freehand, cross-sections were made at distances of 10, 30, 50, and 70mm from the root apex. To detect the suberin lamellae (SLs) in the endo- and exodermis, which are known to be the barriers for nutrient transport, cross-sections were stained with the lipophilic fluorochrome fluorol yellow 088 (Brundrett et. al., 1991). Sections were viewed under an epifluorescence microscope using an ultraviolet filter set (excitation filter BP 365, dichroic mirror FT 395, barrier filter LP 397; Zeiss, Oberkochen, Germany).

### Root pressure probe measurements

Root pressure probe measurements were carried out for excised end segments of roots lacking laterals, as previously described by Miyamoto *et al.* (2001). Healthy roots of WT and *OsAMT1;1* transgenic lines (L-2 and L-3) were selected from the plants grown in optimum nitrogen (300 μM). The average length of measured root segments ranged from 60mm to 90mm. Using cylindrical seals prepared from liquid silicone material (Xantopren; Bayer, Leverkusen, Germany), excised end segments of individual roots (i.e. tips intact) were tightly but carefully connected to a root pressure probe without damaging them. Usually, it took 4–8h to establish stable root pressures.

Once roots attained stable root pressures, the nutrient solution in the medium was rapidly exchanged with a medium containing 25mM (NH_4_)_2_SO_4_ (∼75 mOsmol kg^–1^ or 0.1875MPa in osmotic pressure), which is a permeating solute. The external root medium was rapidly bubbled with air to mix the solution and to minimize the effects of the external, unstirred layers. The changes in root pressure in response to changes in the osmotic pressure of the medium were biphasic. There was a rapid water phase (water efflux or influx) followed by a slower solute phase (solute permeation into or out of the root; Supplementary Fig. S2 available at *JXB* online). The solute phase of the biphasic pressure relaxation was used to calculate the permeability coefficient of (NH_4_)_2_SO_4_ (*P*_sr_ in m s^–1^) as earlier described by Steudle *et al.* (1987):

$$k_{sr}\;=\;\frac{\ln(2)}{t_{1/2}^{s}}\;=\;\frac{A_{r}\;\times \;P_{sr}}{V_{x}}\;,$$ (1)

where *k*_sr_ is the rate constant of permeation of (NH_4_)_2_SO_4_ or the half-time of solute exchange (*t* ^s^_1/2_) and *V*_x_ is the volume of mature xylem. *V*_x_ was 1–2% of the total root volume (depending on the root used; Miyamoto *et al.*, 2001). Cutting experiments were conducted to validate the readings after each experiment. When the xylem of the root remained open, there was a rapid drop in root pressure to zero. If this did not occur, the results were discarded. Ten replicates were used for each line.

### Analysis of NH_4_^+^ uptake into the xylem

To determine the direct fluxes of NH_4_^+^ into the xylem and its dynamics, 1-month-old transgenic and WT plants grown in optimum nitrogen (300 μM NH_4_^+^) were transferred to the nutrient solution with different levels of nitrogen (30, 100, 300, 1000, 2000, or 3000 μM NH_4_^+^). One hour later, shoots were cut off using razor blades at distances of 40–70mm from their base. All except the main stem were closed using clamps. Using a microsyringe, xylem sap exuding from the cut surface of the main stem was collected continuously in Eppendorf tubes for 30min and weighed. The amount of NH_4_^+^ in the xylem sap was determined using the Megazyme kit according to the manufacturer’s instructions (Megazyme International Ireland Ltd, Wicklow, Ireland). The NH_4_^+^ uptake rates and dynamics were determined by normalizing its concentration to the time xylem sap was collected and to root surface area (μmol m^–2^ s^–1^). The NH_4_^+^ flux or uptake rate in the xylem was plotted against the NH_4_^+^ concentration in the medium. Four to six replicates were used for each treatment.

To determine the increase of NH_4_^+^ concentration in the xylem sap with time, which is directly linked to the functional transport protein level as well as radial NH_4_^+^ transport in roots, WT and transgenic plants (L-2 and L-3) were grown in optimum nitrogen (300 μM NH_4_^+^) for a month. As described earlier, the main tiller was severed with a razor blade and the exuded xylem sap was collected from the cut surface every 15min for 75min with the aid of a microsyringe. The concentration of NH_4_^+^ in the xylem sap was determined using the Megazyme kit and normalized to the root surface area (μmol m^–2^). The NH_4_^+^ concentration was then plotted against time. All measurements were done in 300 μM NH_4_^+^. Four to six replicates were used for each treatment.

### Ammonium and glutamine analysis

To quantify ammonium and glutamine, young, fully expanded leaves and healthy adventitious roots were frozen in liquid nitrogen and ground to a fine powder. Samples were then freeze-dried overnight. A 20mg aliquot of freeze-dried sample was dissolved in 400 μl of 100% methanol by incubating on a shaker at 1400rpm at 25 ºC for 10min. The sample was centrifuged at 13 000rpm for 5min and the supernatant was transferred to a mixture of water and chloroform. The top layer of water and methanol without chloroform with chlorophyll (the bottom layer) was separated, and freeze-dried. Ammonium and glutamine analysis was performed using the Megazyme kit according to the manufacturer’s instructions (Megazyme International Ireland Ltd). All measurements were quantified by colorimetric 96-well plate assays using a standard spectrophotometer (MULTISKAN GO, Thermo Fisher Scientific, Vantaa, Finland). Five replicates were used for each treatment.

### Chlorophyll and carotenoid measurements

The standard acetone extraction method was used to extract chlorophyll from fresh leaf tissues of plants grown under low, optimum, and high NH_4_^+^ levels for 4 weeks (Sestak, 1971). A 100 μg aliquot of fresh weight was used in each sample to extract chlorophyll, incubating the samples in 80% acetone for 15min in the dark. Samples were centrifuged at 12,000rpm for 1min and the supernatants were transferred to new vials. Chlorophyll extraction was continued until the pellets became white, at which stage no chlorophyll was left in the sample. The absorbance of the supernatant was measured at *A*_663_, *A*_645_, and *A*_480_ nm, using a standard spectrophotometer (MULTISKAN GO, Thermo Fisher Scientific). Eighty percent acetone was used as the blank. Four replicates were used for each treatment.

### Starch, sucrose, and glucose analysis

For starch and sugar quantification, young, fully expanded leaves were frozen in liquid nitrogen and ground to a fine powder. Samples were then freeze-dried overnight. A 300mg aliquot of freeze-dried sample was extracted with 1ml of 100% methanol by shaking at 50 ºC for 15min. The extraction was repeated five times and insoluble residue was weighed. Then 20mg of the extracted sample were used for starch and sugar (sucrose and glucose) analysis using the Megazyme kit according to the manufacturer’s instructions (Megazyme International Ireland Ltd). To determine the starch content of seeds, 20mg of dried and dehusked seeds were used. Seeds were crushed into powder and extracted with 1ml of 100% methanol five times as described earlier. Five replicates were used for each treatment.

### Yield study with different NH_4_^+^ dosages

All plants were harvested at maturity, and panicles from each plant with their stems were bagged separately. The total numbers of filled and empty spikelets (grains) were counted, and total grain yield was measured for each WT and transgenic plant. Twenty-four plants from each line (either WT or transgenic) were used for yield analysis.

### Statistical analysis

Data were normally distributed and presented as the means ±SD. Data analysis was done using analysis of variance (ANOVA) and the means were compared using the least significant difference (LSD) test at the *P* ≤ 0.05 level. The analytical software Statistix was used to perform all statistical tests.

## Results

### Basic phenotypic parameters and plant biomass contents from the overexpression of *OsAMT1;1* in rice

**Fig. 1.**
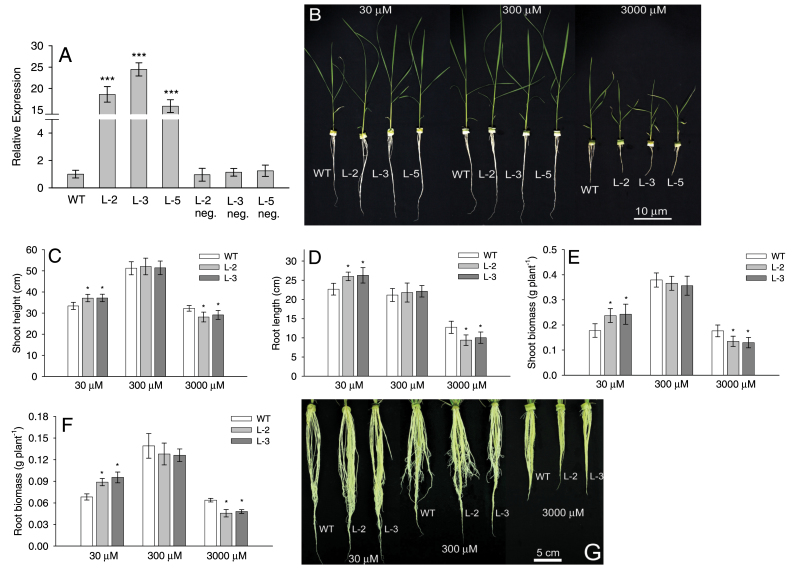
The relative expression of the *OsAMT1;1* gene and plant growth parameters of transgenic and wild-type plants grown in hydroponics. (A) Quantitative real-time PCR analysis of *OsAMT1;1* gene expression in transgenic lines, wild-type, and azygous control plants, grown under optimum NH_4_^+^ (300 μM). (B) Phenotypes of 2-week-old transgenic lines (L-2, L-3, and L-5) together with wild-type plants, grown under different NH_4_^+^ levels (30, 300, and 3000 μM), and their basic parameters: (C) shoot heights, (D) root lengths, (E) shoot biomass, and (F) root biomass. (G) Phenotypes of roots grown at different NH_4_^+^ levels (30, 300, and 3000 μM) for 4 weeks. Significance levels of *P* ≤ 0.001 or *P* ≤ 0.05 are denoted by either *** or *, respectively (ANOVA, LSD test). Data are means ±SD of 12 replicates.

The relative expression levels of the *OsAMT1;1* gene, quantified by qRT-PCR, were significantly greater in the transgenic lines than in the WT. On average, the transgenic lines showed 15- to 25-fold the expression of WT and azygous control plants (Fig. 1A). Among the transgenic lines, the highest expression level was detected in the L-2 and L-3 lines (Fig. 1A). Therefore, these two lines were selected for further experimental analysis.

When grown in different NH_4_^+^ levels, rice plants at 300 μM delivered the maximum growth in both WT and transgenic plants, indicating that of the concentrations tested it is optimal for rice plant growth and development (Fig. 1B). In contrast, 30 μM NH_4_^+^ produced nitrogen-deficient symptoms in plants, whereas 3000 μM produced NH_4_^+^ toxicity and poor plant growth (Fig. 1B). Hence, 300 μM NH_4_^+^ was set as the optimum nitrogen concentration and also denoted as the control condition. In this concentration, the growth performances of WT and transgenic plants were the same (Fig. 1B–F). When grown in 30 μM NH_4_^+^, in general plants performed worse than the controls, showing shorter shoots and lower shoot and root biomass levels (Fig. 1B–F). However, under this nitrogen-deficient condition, transgenic plants performed better than WT plants, exhibiting significantly longer shoots and roots, and greater shoot and root biomass (*P* < 0.05). When grown in high nitrogen, 3000 μM NH_4_^+^, transgenic and WT plants showed poor growth compared with the control (300 μM), probably due to NH_4_^+^ toxicity. This poor growth included shorter shoots and roots, and lower shoot and root biomass than the controls (Fig. 1C–F). However, in the higher NH_4_^+^ concentration, the WT performed better than the transgenic lines, which showed severe growth-retarded symptoms due to excessive uptake and accumulation of NH_4_^+^. In this concentration, WT plants had significantly longer shoots and roots, as well as greater shoot and root biomass than transgenic lines (*P* < 0.05; Fig. 1C–F). Azygous control plants in which progeny from the transgenic lines did not carry the transgene were identified. Comparison of these plants (L-2 neg. and L-3 neg.) with the WT showed that they all behaved in the same way (Supplementary Fig. S3A–E available at *JXB* online). Root systems of 4-week-old mature plants, grown in different NH_4_^+^ levels, clearly showed that the transgenic lines performed better than the WT at limiting ammonium levels, while the converse was found under toxic levels of ammonium (Fig. 1G).

### Relative expression of the *OsAMT1;1* gene in shoots and roots

**Fig. 2.**
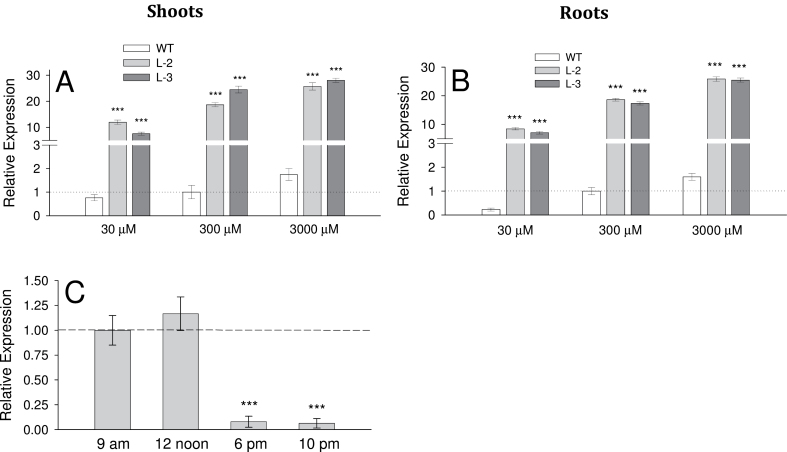
(A, B) The relative expression of the *OsAMT1;1* gene in shoots and roots of transgenic lines and wild-type plants grown under different NH_4_^+^ levels (30, 300, and 3000 μM) quantified by qRT-PCR. (C) Expression of the *OsAMT1;1* gene in leaves of wild-type plants at different times of the day. A significance level of *P* ≤ 0.001 is denoted by *** (ANOVA, LSD test). Data are means ±SE of three biological and three technical replicates.

For comparison, the expression of the *OsAMT1;1* gene in WT plants grown in 300 μM NH_4_^+^ (optimum concentration) was set as the base value of 1 in both roots and shoots. The relative expression of the *OsAMT1;1* gene in transgenic lines was ~10- to 30-fold the expression in the WT, in both roots and shoots (*P* ≤ 0.001: Fig. 2A, B). In general, plants grown in 3000 μM exhibited the highest expression levels, whereas plants in 30 μM exhibited the lowest expression. The expression of plants at 300 μM was intermediate (Fig. 2A, B). This indicates that *OsAMT1;1* is an NH_4_^+^-responsive gene. Analysis of relative *OsAMT1;1* gene expression throughout the day showed that its expression was greatest from morning to noon, but decreased significantly in the late afternoon and evening (Fig. 2C). This was an ~8-fold reduction compared with its maximum value at noon.

### Cellular localization of the high-affinity *OsAMT1;1* ammonium transporter gene

**Fig. 3.**
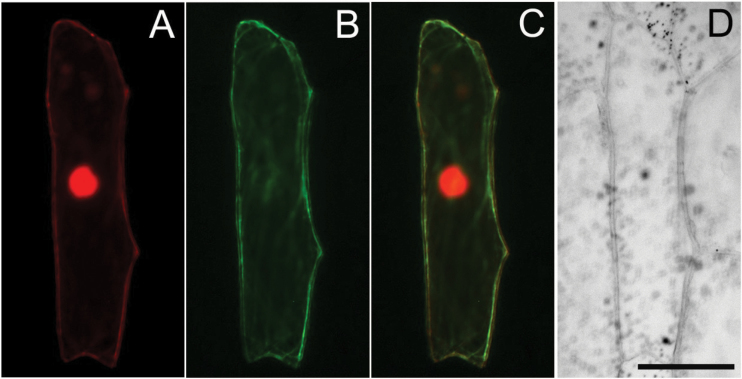
Intercellular localization of OsAMT1;1 protein, investigated in onion epidermal cells expressing GFP::OsAMT1;1 fusions together with plasma membrane-localized red fluorescent protein (pRTL2-RFP ARC3). (A) RFP fused to the plasma membrane in successfully transformed onion epidermal cells. (B) GFP-dependent fluorescence in the plasma membrane of onion epidermal cells. (C) Merged picture of A and B showing co-localization of RFP and GFP. (D) Brightfield micrograph of the onion epidermal cell. Bar=50 μM.

The intercellular localization of the *OsAMT1;1* gene was investigated in onion epidermal cells expressing N-terminal GFP::OsAMT1;1 fusions under the control of the 35S promoter from *Cauliflower mosaic virus* (CaMV). As further confirmation of successful transformation, a plasma membrane-localized RFP marker (pRTL2-RFP RAC3) was also used for biolistic bombardment. Successfully transformed cells exhibited RFP fused to the plasma membrane in the onion epidermis (Fig. 3A). GFP-dependent fluorescence was also observed in the plasma membrane of onion epidermal cells (Fig. 3B). The merged image (Fig. 3C) clearly showed co-localization of RFP and GFP in the plasma membrane; Fig. 3D shows a light micrograph of the transformed onion epidermal cell. These results indicated that the OsAMT1;1 transporter protein is localized in the plasma membrane.

### Relative expression of genes involved in the nitrogen assimilation pathway

**Fig. 4.**
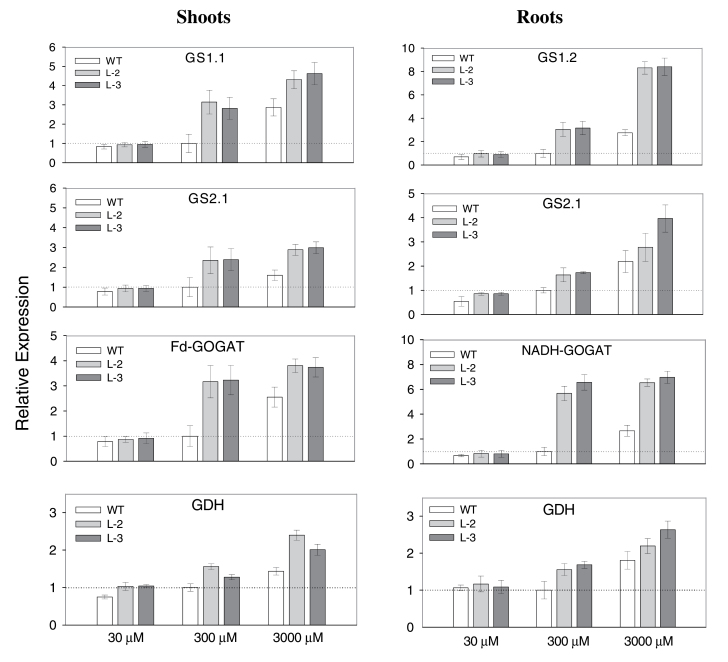
Relative expression of genes involved in the nitrogen assimilation pathway quantified by qRT-PCR: cytosolic glutamine synthetase (*GS1.1*), chloroplastic glutamine synthetase (*GS2.1*), Fd-glutamate synthase (*Fd-GOGAT*), and glutamate dehydrogenase (*GDH*) in shoots; and *GS1.2*, *GS2.1*, *NADH-GOGAT*, and *GDH* in roots, was analysed after transferring plants to a hydroponic system with different levels of NH_4_^+^ (30, 300, or 3000 μM) for 2h, and the wild type grown in 300 μM was set as the control. Data are means ±SE of three biological and three technical replicates.

The expression levels of genes involved in the nitrogen assimilation pathway, such as *GS1.1*, *GS2.1*, *Fd-GOGAT*, and *GDH* in shoots as well as *GS1.2*, *GS2.1*, *NADH-GOGAT*, and *GDH* in roots, were analysed in plants after transferring them to a hydroponic system with different levels of nitrogen (30, 300, or 3000 μM NH_4_^+^). In general, the highest expression levels of nitrogen assimilation genes, in both shoots and roots, were observed in plants grown at higher (3000 μM) NH_4_^+^ levels, whereas the lowest levels were seen in plants grown at low (30 μM) NH_4_^+^ levels (Fig. 4). At 300 μM, which was set as the optimum, the expression level was moderate. Comparison of transgenic lines with the WT showed a significantly higher expression of genes in the nitrogen assimilation pathway in transgenic plants than in the WT in all growth conditions with different NH_4_^+^ levels, except for the suboptimal level of 30 μM (Fig. 4). Therefore, the expression of these genes appears to be directly linked to the NH_4_^+^ concentration in the plant, with higher levels present in WT plants grown under higher NH_4_^+^ conditions or in transgenic lines overexpressing the ammonium transporter.

### Comparison of root structures

**Fig. 5.**
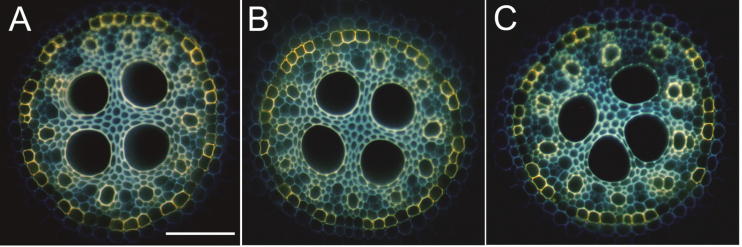
Structural comparison of rice roots. Development of suberin lamellae in the endodermis of wild-type (WT) and transgenic lines (L-2 and L-3) grown in hydroponic solution with optimum NH_4_^+^ (300 μM) for 4 weeks, and stained with fluorol yellow 088. The presence of suberin lamellae is indicated by bright yellow fluorescence in the endodermis. At 50mm, half of the endodermal cells had suberin lamellae in the WT (A) and transgenic lines (B, C). Bar=50 μm.

There were no structural differences in roots between WT and transgenic (L-2 and L-3) plants (Fig. 5). The SL, a known barrier for nutrient transport in roots, can be detected as an intense, bright yellow fluorescence by staining with the lipophilic fluorochrome, fluorol yellow 088. At 50mm from the apex, patchy or discontinuous SL were present in the endodermis of all roots of WT and transgenic lines (Fig. 5). The same was found in the exodermis (data not shown).

### Root NH_4_^+^ uptake and xylem transport in WT and transgenic plants

**Fig. 6.**
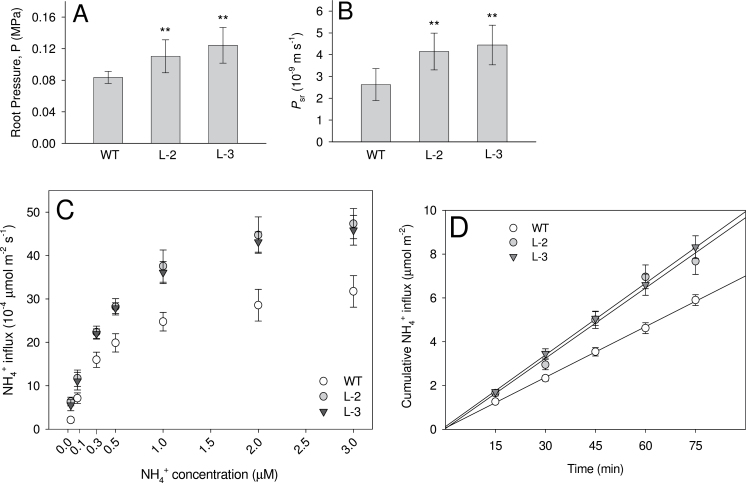
Permeability measurements of rice plants grown in hydroponics for 4 weeks. (A) Steady-state root pressures (*P*_r_) and (B) solute permeability (*P*_sr_) of *OsAMT1;1* transgenic (L-2 and L-3) and wild-type (WT) roots for (NH_4_)_2_SO_4_ measured with a root pressure probe. (C) Dynamics of NH_4_^+^ uptake or fluxes of the whole root systems, analysed from the NH_4_^+^ concentration in the xylem sap, in response to different NH_4_^+^ levels in the medium. (D) Cumulative NH_4_^+^ concentration in the xylem plotted against time, measured under the optimum NH_4_^+^ (300 μM) level. Ten plants from each line were used to measure *P*_r_ and *P*_sr_, while 4–6 plants from each line were used to measure NH_4_^+^ in the xylem. Significance levels of *P* ≤ 0.001 or *P* ≤ 0.01 are denoted by either *** or **, respectively (ANOVA, LSD test).

The steady-state root pressures (*P*_r_s), measured with a root pressure probe, indicate the capacity for ion uptake by roots and accumulation of those ions in the root xylem. Analysis of the *P*_r_ of WT and *OsAMT1;1* transgenic lines showed that both transgenic lines had significantly greater *P*_r_ than WT plants (*P* < 0.05; Fig. 6A). The non-transgenic lines had the same *P*_r_ as the WT (Supplementary Fig. S4A available at *JXB* online). The difference in root pressure between the WT and transgenic lines was probably due to the greater uptake of NH_4_^+^ in transgenic lines compared with the WT.

There was a marked difference between WT and transgenic roots in the solute permeability (*P*_sr_) of (NH_4_)_2_SO_4_. *P*_sr_ of transgenic roots was significantly (1.7- to 2-fold) greater than in WT roots (*P* < 0.01; Fig. 6B). The average *P*_sr_ was 2.6, 4.2, and 4.4×10^–9^ m s^–1^ for WT and transgenic (L-2 and L-3) roots, respectively. The non-transgenic lines behaved like the WT and their *P*_sr_ values for (NH_4_)_2_SO_4_ were the same (Supplementary Fig. S4B available at *JXB* online). The measured *P*_sr_ for K_2_SO_4_ was the same for the WT and all transgenic lines (data not shown). This indicated that the difference in *P*_sr_ between transgenic lines and the WT was due to different NH_4_^+^ permeabilities.

Direct functional uptake was determined from the NH_4_^+^ flux into the xylem, and the transgenic lines had ~1.5- to 3-fold greater values than the WT, depending on the NH_4_^+^ concentration applied in the medium. Furthermore, irrespective of the plant type (either WT or transgenic lines), the rate of NH_4_^+^ uptake was increased with increasing NH_4_^+^ concentration in the medium. However, there was an apparent trend of saturation of NH_4_^+^ uptake or fluxes in the roots between 1000 μM (1mM) and 2000 μM (2mM) levels in the medium (Fig. 6C). The level of saturation was significantly greater in the transgenic plants than in the WT.

The increase of NH_4_^+^ concentration in the xylem sap with time, which directly links to the functional transport protein level in the roots, showed that transgenic plants had significantly greater uptake rates or fluxes than the WT (Fig. 6D). The cumulative NH_4_^+^ concentration (μmol) in the xylem increased linearly with time in both transgenic and WT plants. However, the transgenic plants had higher values at all time points tested.

### NH_4_^+^ and glutamine concentration in shoots and roots

**Fig. 7.**
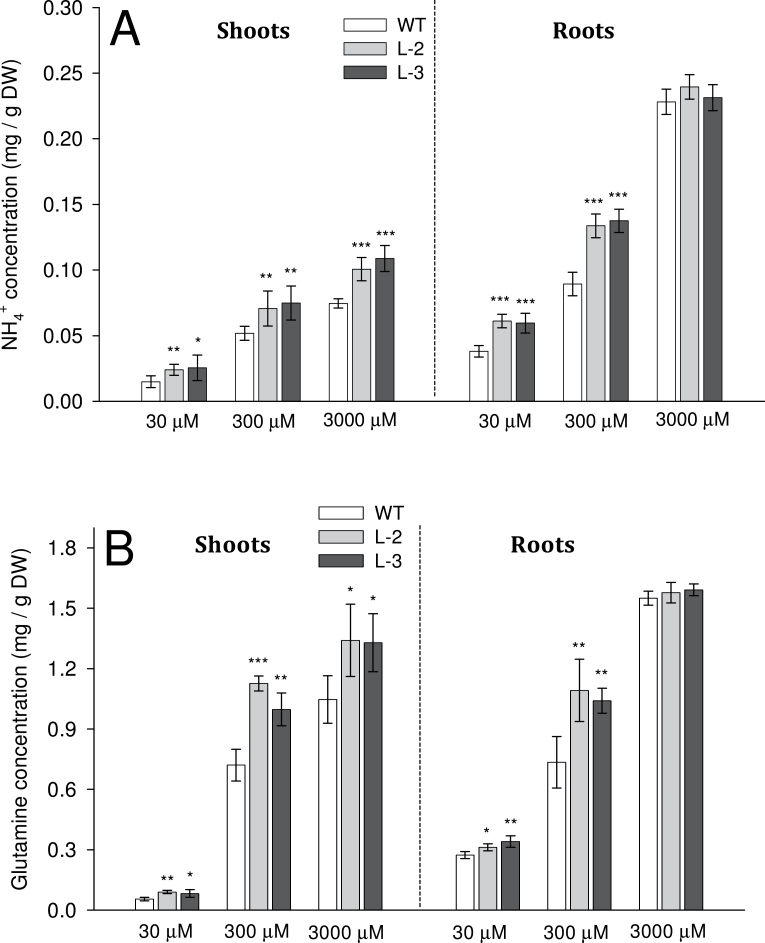
NH_4_^+^ and glutamine concentrations in shoots and roots of rice plants grown in hydroponics for 4 weeks. (A) Total NH_4_^+^ and (B) glutamine concentrations in shoots and roots of plants grown under different NH_4_^+^ levels (30, 300, or 3000 μM) in the medium, measured with a spectrophotometric method. Significance levels of *P* ≤ 0.001 or *P* ≤ 0.01 are denoted by either *** or **, respectively (ANOVA, LSD test). Data are means ±SD of five replicates.

Analysis of total NH_4_^+^ in rice shoots and roots demonstrated that transgenic plants had significantly greater amounts than in the WT, grown in all growth conditions, except for roots grown in 3000 μM (*P* < 0.05; Fig. 7A). In general, roots had a higher NH_4_^+^ concentration than that found in shoots (Fig. 7A). Plants grown at 30 μM had the lowest values, whereas plants grown at 3000 μM had the greatest values. Again, it was intermediate for plants grown at the 300 μM NH_4_^+^ level. The non-transgenic lines behaved like the WT, and the total NH_4_^+^ concentration was the same for both plant lines (Supplementary Fig. S5A available at *JXB* online).

In general, glutamine concentrations in the shoots and roots were many fold greater than the NH_4_^+^ concentration in the respective tissues (Fig. 7A, B). The values of total glutamine of transgenic lines were significantly greater than that of the WT, except for roots grown at 3000 μM NH_4_^+^ (*P* < 0.05; Fig. 7B). Plants grown in 30 μM NH_4_^+^ had the lowest values, whereas plants grown in 3000 μM had the highest values in both WT and transgenic plants (Fig. 7B). When grown at optimum NH_4_^+^, azygous control lines had significantly lower concentrations of glutamine than their respective positive lines and behaved like WT plants (*P* < 0.05; Supplementary Fig. S5B available at *JXB* online).

### Photosynthetic pigments, starch, and sucrose concentrations in WT and transgenic plants

**Fig. 8.**
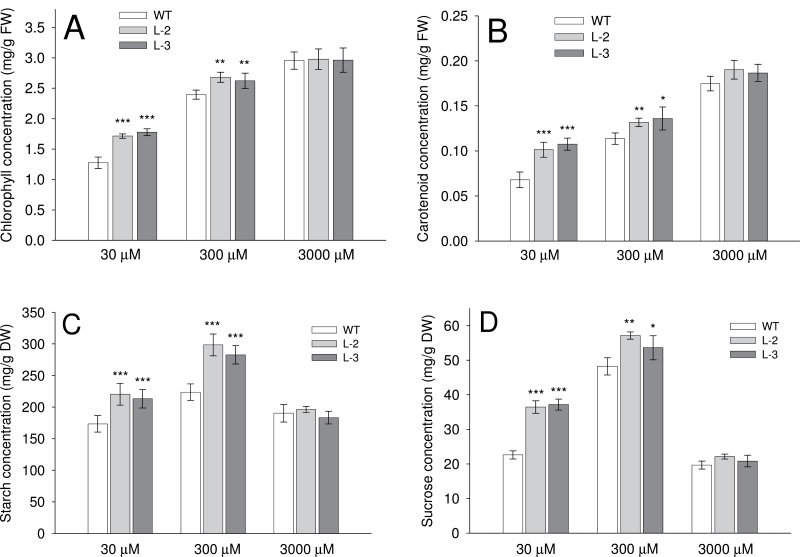
Comparison of photosynthetic pigments, sugars, and starch of plants grown in hydroponics for 4 weeks. (A) Total chlorophyll, (B) carotenoids, (C) starch, and (D) sucrose concentrations in the leaves of wild-type (WT) and *OsAMT1;1* transgenic (L-2 and L-3) plants, grown in different levels of NH_4_^+^ (30, 300, or 3000 μM), measured with a spectrophotometric method. Data are means ±SD of four replicates for chlorophyll and carotenoids, and of five replicates for starch and sucrose. Significance levels of *P* ≤ 0.001 or *P* ≤ 0.01 are denoted by either *** or **, respectively (ANOVA, LSD test).

Analysis of photosynthetic pigments in leaves, such as total chlorophyll (*a*+*b*) and carotenoids, revealed that transgenic lines had significantly greater values than WT plants, grown at both low (30 μM) and optimum (300 μM) NH_4_^+^ levels (*P* < 0.05; Fig. 8A, B). It was analogous to the NH_4_^+^ concentration in the plant, in that transgenic lines had greater uptake and assimilation compared with the WT. In contrast, plants grown in higher NH_4_^+^ (3000 μM) had no significant difference between the WT and transgenic lines (Fig. 8A, B), and all showed a ‘super green’ phenotype. Analysis of starch and sucrose in leaves showed that both transgenic and WT plants achieved the greatest values when grown at optimum NH_4_^+^ levels (300 μM; Fig. 8C, D). Plants grown in 3000 μM exhibited the lowest values, while they were intermediate for 30 μM, for both WT and transgenic plants (Fig. 8C, D). In general, the total glucose concentration in leaves was significantly lower than that of starch and sucrose but exhibited the same trend as the latter products in all plants grown at different levels of NH_4_^+^ (Supplementary Fig. S6A available at *JXB* online). The highest NH_4_^+^ level used, 3000 μM, was toxic and thus probably also inhibited photosynthesis. At both 30 μM and 300 μM, transgenic plants had significantly higher starch and sucrose concentrations in leaves than their WT counterparts (*P* < 0.05; Fig. 8C, D), which was positively linked to the greater values of photosynthetic pigments such as chlorophyll and carotenoids. In contrast, the starch concentration of rice seeds was the same for WT and transgenic plants grown at different levels of NH_4_^+^ (Supplementary Fig. S6B available at *JXB* online).On average, the values of seed starch were many fold greater than the values of leaf starch in all plants.

### Number of panicles, total spikelets, and grain yield in WT and transgenic plants

**Fig. 9.**
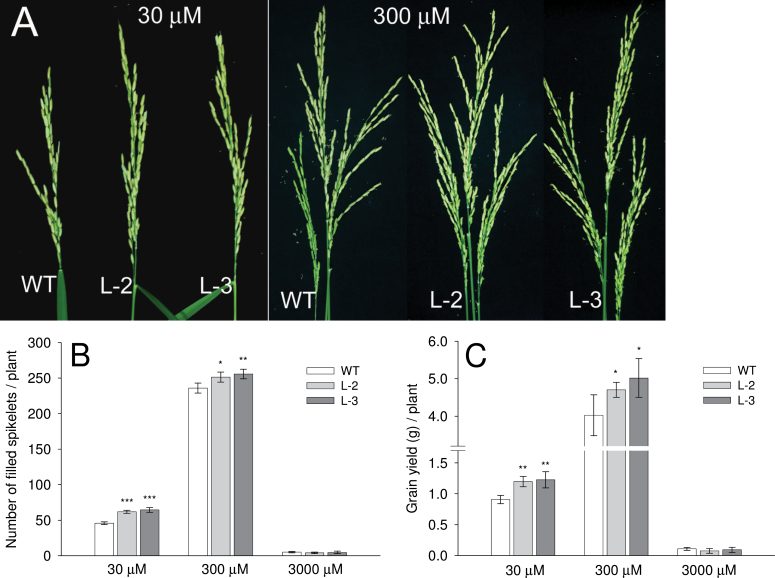
Comparison of yield in *OsAMT1;1* transgenic (L-2 and L-3) and wild-type (WT) plants. (A) Panicle morphology, (B) filled grains or spikelets, and (C) total grain yield of plants grown in different levels of NH_4_^+^ (30, 300, or 3000 μM) in the medium. Data are means ±SD of 24 plants. Significance levels of *P* ≤ 0.001, *P* ≤, 0.01 or *P* ≤ 0.05 are denoted by either by ***, **, or *, respectively (ANOVA, LSD test).

When grown in optimum nitrogen (300 μM NH_4_^+^), transgenic and WT plants had three panicles per plant (Fig. 9A), though the total number of spikelets was significantly greater in transgenic lines than in the WT (*P* < 0.05; Supplementary Fig. S7A available at *JXB* online). Suboptimal nitrogen (30 μM NH_4_^+^) resulted in the development of one panicle per plant in both the WT and transgenic lines (Fig. 9A). However, as observed for optimum nitrogen, the total number of spikelets was significantly greater in transgenic plants (*P* < 0.05; Supplementary Fig. S7A available at *JXB* online). High nitrogen (3000 μM NH_4_^+^) delayed flowering in all plants for a month compared with optimum and low nitrogen levels. In this high concentration, both plants produced 3–4 panicles per plant (data not shown). The number of filled spikelets was significantly higher in transgenic lines than in the WT, at both low and optimum NH_4_^+^ levels (*P* < 0.05; Fig. 9B). In contrast, there was a dramatic reduction of filled grains in all plants under high NH_4_^+^ which resulted in >95% empty grains (Fig. 9B; Supplementary Fig. S7B available at *JXB* online). In summary, the grain yield increased in transgenic lines by >30% and 20% per plant compared with the WT under low and optimum nitrogen levels, respectively (Fig. 9C).

## Discussion

In paddy rice, the bulk of the soil is hypoxic or anoxic and the major source of nitrogen available to plants is ammonium ions (NH_4_^+^). NH_4_^+^ is also the preferred nitrogen source taken up by rice and it is superior to nitrate (Yoshida, 1981; Kronzucker *et al.*, 2001). Thus, NH_4_^+^ transporters in roots may be expected to be crucial for nitrogen uptake in rice plants. In this study, the effect of the overexpression of the *OsAMT1;1* gene on radial NH_4_^+^ uptake in rice roots was studied. For the first time, the physiological measurements obtained demonstrate that the NH_4_^+^ transporter gene, *OsAMT1;1*, a nitrogen-responsive member of the *AMT1* gene family, plays a significant role in radial NH_4_^+^ uptake in rice roots as well as subsequent NH_4_^+^ transport into the shoot. This increase in the level of *OsAMT;1* expression can also have positive and negative effects on rice growth, depending on the NH_4_^+^ concentration in the root medium.

When measured with the root pressure probe, the *OsAMT1;1* transgenic plants had a significantly higher steady-state root pressure (*P*_r_) than the WT. This is probably due to enhanced uptake of NH_4_^+^ ions into the root xylem (Fig. 6A). However, despite large net ammonium uptake in transgenic plants, there should also be a considerable efflux from roots to the medium (Britto *et al.*, 2001), mainly through the less selective apoplast. The higher *P*_r_ in transgenic rice plants positively correlated with the 2-fold greater NH_4_^+^ permeability in roots (*P*_sr_) compared with the WT (Fig. 6B). To confirm that this permeability difference between the transgenic and WT plants was solely due to NH_4_^+^ ions, and not due to differences in the permeability of SO_4_^2–^ ions, permeability measurements were carried out using K_2_SO_4_. The permeability coefficient of roots (*P*_sr_) for K_2_SO_4_ was the same for *OsAMT1;1* transgenic and WT plants (data not shown). This confirms that the increase in *P*_sr_ of (NH_4_)_2_SO_4_ in transgenic plants was due to higher NH_4_^+^ transport across the roots.

The pressure probe measures NH_4_^+^ flux through all pathways into the root. Generally, nutrient ions move radially across roots from the root medium to the xylem through the apoplastic (cell walls) and cell–cell paths. The latter includes the transport of ions through the plasma membrane as well as through the plasmodesmata (Steudle and Peterson, 1998). The relative contribution of each pathway for ion movement can be varied depending on the resistances along the paths. Anatomical studies showed that there are no apparent root structural differences between the WT and transgenic lines (Fig. 5). Therefore, the transport measured through the pathways in roots does not involve an alteration of the NH_4_^+^ transporter in the transgenic plants. Thus, it is not surprising that the transgenic plants only had 1.5- to 3-fold greater fluxes depending on the applied NH_4_^+^ concentration in the medium (Fig. 6C).

The relative gene expression levels of the other members of the *AMT* family (*OsAMT1;2* and *OsAMT1;3*) in roots are the same for transgenic and WT plants grown under an optimum NH_4_^+^ level (data not shown). Hence, the greater NH_4_^+^ uptake in transgenic lines is most probably due to higher NH_4_^+^ transport through the OsAMT1;1 protein in the plasma membrane. This is in agreement with the study of Yuan *et al.* (2006) in which *AtAMT1;1*-transformed tobacco plants had 30% higher NH_4_^+^ influx into roots compared with the WT. However, these authors determined the NH_4_^+^ uptake per unit root dry weight rather than per unit surface area. On the other hand, *AtAMT1;1* and *AtAMT1;2* mutant lines of *Arabidopsis* had ~30% and 18–26% lower overall ammonium uptake capacity in roots than the WT, respectively (Kaiser *et al.*, 2002; Yuan *et al*., 2007). These studies demonstrate that the numerous members of the *AMT1* family are involved in NH_4_^+^ uptake in roots. However, changes to one member would result in altering overall NH_4_^+^ uptake in roots.

Even though some members of the NH_4_^+^ transporter family in different plant species are known to be located in the plasma membrane, the exact location of the *OsAMT1;1* protein in rice had not been determined. The present GFP localization studies with onion epidermal cells showed that the protein product of the *OsAMT1;1* gene is localized in the plasma membrane (Fig. 3), which is in agreement with the speculation of Sonoda *et al.* (2003) who utilized a hydropathy profiling method to predict the localization of the OsAMT1;1 protein. It is reasonable, based on both the localization studies and the transport study, to assume that this membrane-bound transporter protein facilitates NH_4_^+^ ion movement from cell to cell. In the case of roots, the OsAMT1;1 protein is likely to play a greater role in the endodermis and exodermis where the radial apoplastic path is blocked by the deposition of Casparian bands (Miyamoto *et al.*, 2001; Ranathunge *et al.*, 2003). This is in agreement with the *AtAMT1;2* gene in *Arabidopsis* which is mainly localized in the endodermis (Neuhäuser *et al.*, 2007; Yuan *et al.*, 2012). When the apoplastic path is blocked, NH_4_^+^ ions have to cross these barriers solely through the transmembrane and symplastic (plasmodesmata) pathways, bypassing the barriers, to reach the root xylem. On the other hand, the OsAMT1;2 protein, another member of the OsAMT1 family, is preferentially localized in the central root cylinder (Sonoda *et al.*, 2003). This suggests that this protein plays a role in transporting NH_4_^+^ from the cortex to the vascular system in the central cylinder of roots.

Once NH_4_^+^ ions move into the root xylem they can freely move into the shoot with the xylem flow (Tabuchi *et al.*, 2007). In this study, greater NH_4_^+^ uptake in transgenic roots directly increased the total NH_4_^+^ concentration in roots and shoots, which is in agreement with previous studies (Fig. 7A, B; Hoque *et al.*, 2006). It is plausible that higher NH_4_^+^ concentrations in transgenic plants result in enhanced NH_4_^+^ assimilation. The measured content of glutamine, which assimilates cytosolic and chloroplast NH_4_^+^ with the aid of the GS1 and GS2 enzymes, respectively, was significantly greater in transgenic lines than in the WT in both roots and shoots (Fig. 7B). Since excessive NH_4_^+^ in the plant is known to be toxic, it is likely that, as a strategy, transgenic plants increased expression of the genes in the NH_4_^+^ assimilation pathway in order to convert free NH_4_^+^ ions into non-toxic glutamine (Fig. 4). In the past, it has been proposed that *GS1.2* and *NADH-GOGAT* could be the key players in assimilation of NH_4_^+^ ions in roots, while *GS1.1* and *Fd-GOGAT* are likely to play a greater role in the remobilization and utilization of nitrogen in the shoot of rice plants (Tabuchi *et al.*, 2007). The present data agree with this hypothesis as all these genes in roots and shoots were up-regulated in transgenic lines compared with the WT (Fig. 4). However, the highest expression for each line was found to be in the plants grown under high NH_4_^+^ levels (3000 μM). This strongly suggests that the expression of genes in the nitrogen assimilation pathway is NH_4_^+^ responsive.

**Fig. 10.**
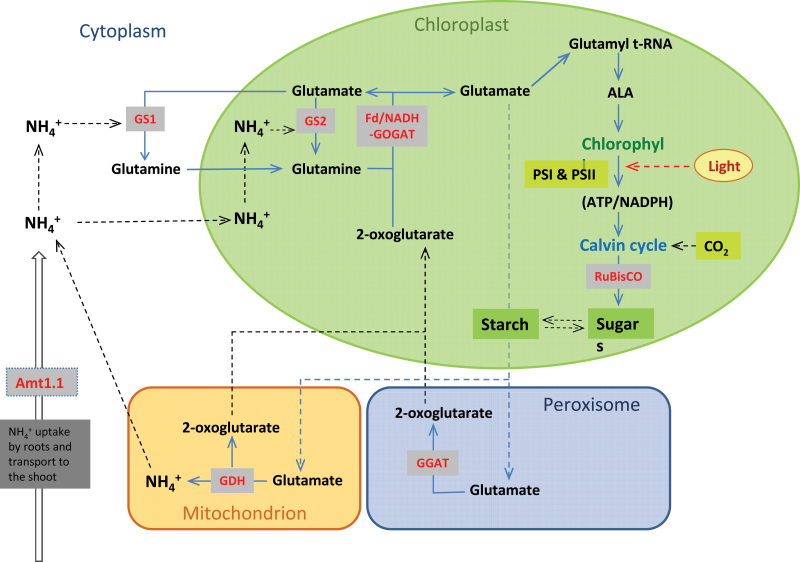
Schematic diagram showing a simplified version of the NH_4_^+^ uptake and assimilation pathway in plants, which ultimately links to carbon assimilation.

It is well known that higher nitrogen assimilation in plants increases the total amino acid content (Lam *et al.*, 1996). However, the correlation between nitrogen assimilation and its ability to improve the carbon metabolism in plants is still not well studied. Coruzzi and Bush (2001) proposed that nitrogen acquisition is intrinsically linked to both photosynthetic activity and the overall carbon status of the plant in a complex and tightly regulated system referred to as the carbon:nitrogen balance. The present data support this view. Higher uptake of NH_4_^+^ in *OsAMT1;1* transgenic plants increased the amount of total nitrogen, thereby promoting photosynthesis and subsequently increased sugar production (Fig. 8). The increment in photosynthesis is probably due to the increased amounts of photosynthesis-related proteins, as earlier described by Makino and Osmond (1991) and Nakano *et al.* (1997). It is also known that the NH_4_^+^ ion is incorporated into a carbon backbone, resulting in production of amino acids, chlorophyll, and other nitrogen-containing biological molecules. In this research, it was obvious that plants with higher NH_4_^+^ levels end up making greater amounts of nitrogenous amino acids, photosynthetic pigments, and subsequently higher levels of sugars. The differences in amino acids, photosynthetic pigments, sugars, and starch between *OsAMT1;1* transgenic plants and the WT was apparent at optimal and suboptimal NH_4_^+^ levels, but was not apparent at high toxic NH_4_^+^ levels. This is probably due to the saturation of enzymes in the nitrogen assimilation pathway under very high NH_4_^+^ levels leading to toxicity effects (Fig. 10). Further, the substantial reduction of sugar and starch contents in all plants grown at the high toxic level of NH_4_^+^ (Fig. 8) is likely to be due to (i) inhibition of RuBisCO enzyme expression and activity due to NH_4_^+^ toxicity; (ii) reduction of CO_2_ uptake in the leaves and lower carbon assimilation due to stomatal closure as a result of osmotic stress in the root medium that created a ‘physiological drought’; and (iii) a block of ATP production in photosystem II of the light reaction and reduction of CO_2_ fixation in the chloroplast (Ikeda and Yamada, 1981).

A seed yield study was carried out comparing the WT with *OsAMT1;1* transgenic overexpression lines in addition to the comparison of growth performances during the vegetative stage (Fig. 9). Enhanced NH_4_^+^ uptake in roots in transgenic plants under suboptimal and optimal levels of (NH_4_)_2_SO_4_ resulted in a significant yield increment with respect to grain filling and total grain yield per plant. This implies that *OsAMT1;1* transgenic rice has the ability to improve NUE, which could reduce the cost of production based on the possibility of using lower application rates of nitrogen fertilizer. However, the addition of very high levels of NH_4_^+^ caused some detrimental effects on the grain-filling process that resulted in extremely low seed setting of all plant lines due to its toxicity. At this NH_4_^+^ concentration, all plant lines exhibited delayed senescence with excessive proliferation of tillers.

In summary, overexpression of the *OsAMT1;1* gene in rice significantly increased the root permeability for NH_4_^+^ ions which resulted in greater nitrogen assimilates, photosynthetic pigments, starch, and sugars than in the WT under low and optimum NH_4_^+^ levels. It also enhanced overall plant growth, especially under low NH_4_^+^ levels, thereby increasing grain filling and total grain yield by >30%. These results suggest that *OsAMT1;1* has the potential for improving NUE under suboptimal (30 μM) and optimal (300 μM) levels of (NH_4_)_2_SO_4_ fertilizer, which is potentially a valuable tool to enhance rice NUE.

## Supplementary data

Supplementary data are available at *JXB* online.

Figure S1. Expression of *OsAMT1;1*, *PMI*, and *Actin-2* genes in wild-type, transgenic (L-2, L-3), and azygous control (L-1 neg., L-2 neg.) plants analysed with RT–PCR.

Figure S2. Time versus pressure change during the osmotic experiment [25mM (NH_4_)_2_SO_4_] with the root pressure probe.

Figure S3. Plant parameters.

Figure S4. Root permeability measurements.

Figure S5. Shoot and root metabolomics.

Figure S6. Glucose and starch measurements

Figure S7. Yield study.

Supplementary Table S1. Sequences of primers used for qRT-PCR to analyse gene expression levels in the nitrogen assimilation pathway.

Supplementary Data
