# Supplementary material for: AMT1;1 transgenic rice plants with enhanced NH4 + permeability show superior growth and higher yield under optimal and suboptimal NH4 + conditions
Source: J Exp Bot. 2014 Jan 13;65(4):965–79. doi: 10.1093/jxb/ert458 (PMC3935567; doi:10.1093/jxb/ert458)
Supplement: Supplementary Data [file supp_65_4_965__index.html]

 AMT1;1 transgenic rice plants with enhanced NH4 + permeability show superior growth and higher yield under optimal and suboptimal NH4 + conditions — Supplementary Data 

# *AMT1;1* transgenic rice plants with enhanced NH4+ permeability show superior growth and higher yield under optimal and suboptimal NH4+ conditions

## Supplementary Data

Data files

**Files in this Data Supplement:**

- Supplementary Data - Supplementary Data
- Supplementary Data - Supplementary Data
