# Supplementary material for: AMT1;1 transgenic rice plants with enhanced NH4 + permeability show superior growth and higher yield under optimal and suboptimal NH4 + conditions
Source: J Exp Bot. 2014 Jan 13;65(4):965–79. doi: 10.1093/jxb/ert458 (PMC3935567; doi:10.1093/jxb/ert458)
Supplement: Supplementary Data [file supp_ert458_ert458_Supplementary_Table_1.pdf]

AMT1;1 transgenic rice plants with enhanced NH_4^+ permeability show superior growth and higher yield under optimal and suboptimal NH_4^+ conditions. *Kosala Ranathunge, Ashraf El-kereamy, Satinder Gidda, Yong-Mei Bi and Steven J. Rothstein*

Supplementary Table S1. Sequences of primers used for qRT-PCR to analyse gene expression levels in the nitrogen assimilation pathway.

Enzyme name	Gene ID	Forward primer (5′ →3′)	Reverse primer (5′ →3′)
GS1.1 (in shoot)	LOC_Os02g50240	CAAGTCCGCCATTGAGAAGC	CTTGCCGTTCTGCTCCGTCT
GS1.2 (in root)	LOC_Os03g12290	GGTTGGAGGATCGGGCATAG	TCACCTTGTGGCGTGTAGCA
GS2.1 (in shoot and root)	LOC_Os04g56400	GGAGCAGGCTGGTGTAGTGC	TCTCCCTGAATTGGTTTGGG
Fd-GOGAT (in shoot)	LOC_Os07g46460	CATCAGGAGGGTTCTGGTGC	CAGGGAAATTGTTTGTCTCAAGT
NADH-GOGAT (in root)	LOC_Os05g48200	AGCCGACCATCACGAGTTTC	CGAGCAGATTGCGTCTTTAGC
GDH (in shoot and root)	LOC_Os01g37760	TGTGACGTTGCATTCCCTTG	CTCCGCCTGGTCAATCTCAT
