# Supplementary material for: AMT1;1 transgenic rice plants with enhanced NH4 + permeability show superior growth and higher yield under optimal and suboptimal NH4 + conditions
Source: J Exp Bot. 2014 Jan 13;65(4):965–79. doi: 10.1093/jxb/ert458 (PMC3935567; doi:10.1093/jxb/ert458)
Supplement: Supplementary Data [file supp_ert458_jexbot115436_file001.pdf]

Supplementary Data:

Figure S1. Expression of *OsAMT1;1*, *PMI* and *Actin-2* genes in wild-type, transgenic (L-2, L-3) and azygous control (L-1 neg., L-2 neg.) plants analyzed with RT-PCR.

Figure S2. Time *verses* pressure change during the osmotic experiment [25 mM $(NH_4)_2SO_4$] with the root pressure probe. The adventitious root was from wild-type (WT) grown in hydroponics for 4 weeks with optimum (300 μM) NH_4^+ level. Addition of 25 mM $(NH_4)_2SO_4$ to the medium resulted in biphasic responses of root pressure; a rapid efflux of water followed by a relatively slow influx of $(NH_4)_2SO_4$ into the root (denoted as solute phase in the graph).

Figure S3. Plant parameters: A, phenotypes of 2-week-old transgenic lines (L-1and L-2) along with the wild-type (WT) and azygous control plants (L-1 neg. and L-2 neg.), grown in different NH_4^+ levels (30, 300, 3000 μM), and their basic parameters; B, shoot heights, C, root lengths, D, shoot biomass, E, root biomass. Significance level of $P \leq 0.05$ is denoted * (ANOVA, LSD test). Data are means ±SD of twelve replicates.

Figure S4. Root permeability measurements: A, steady-state root pressures (P_r) and B, solute permeability (P_{sr}) of transgenic (L-1 and L-2) wild-type (WT) and azygous control (L-1 neg. and L-2 neg.) roots for $(NH_4)_2SO_4$ measured with a root pressure probe.Ten plants from each line were used to measure P_r and P_{sr}.Significance level of $P \leq 0.01$ is denoted by ** (ANOVA, LSD test).

Figure S5. Shoot an root metabolomics: A, total NH_4^+ and B, glutamine concentrations in shoots and roots of transgenic (L-1 and L-2) wild-type (WT) and azygous control (L-1 neg. and L-2 neg.) plants grown different NH_4^+ levels (30 or 300 or 3000 μM) in the medium.Significance levels of $P \leq 0.001$ or $P \leq 0.01$ are denoted either by *** or **, respectively (ANOVA, LSD test). Data are means ±SD of five replicates.

Figure S6. Glucose and starch measurements: A, leaf glucose and B, seed starch concentrations of wild-type (WT) and transgenic (L-1 and L-2) plants, grown in different levels of NH_4^+ (30 or

300 or 3000 μM). Data are means ±SD of five replicates. Significance levels of $P \leq 0.01$is denoted by ** (ANOVA, LSD test).

Figure S7. Yield study: A, total number of grains or spikeletes and B, empty grains per plant of wild-type (WT) and transgenic (L-1 and L-2) plants, grown in different levels of NH_4^+ (30 or 300 or 3000 μM) in the medium. Data are means ±SD of twenty four plants. Significance levels of $P \leq 0.01$ or $P \leq 0.05$ are denoted either by ** or *, respectively (ANOVA, LSD test).

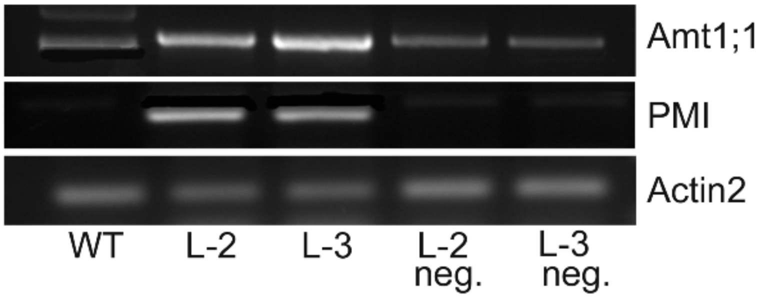

Supplementary Fig. 1

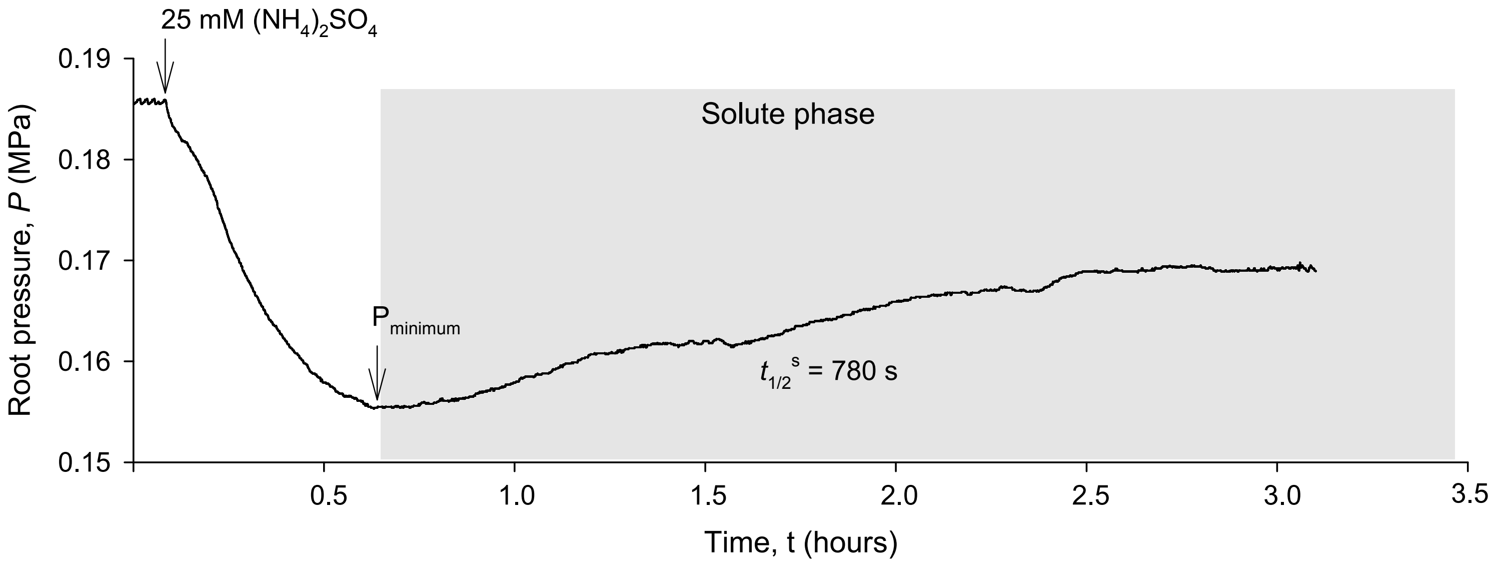

Supplementary Fig. 2

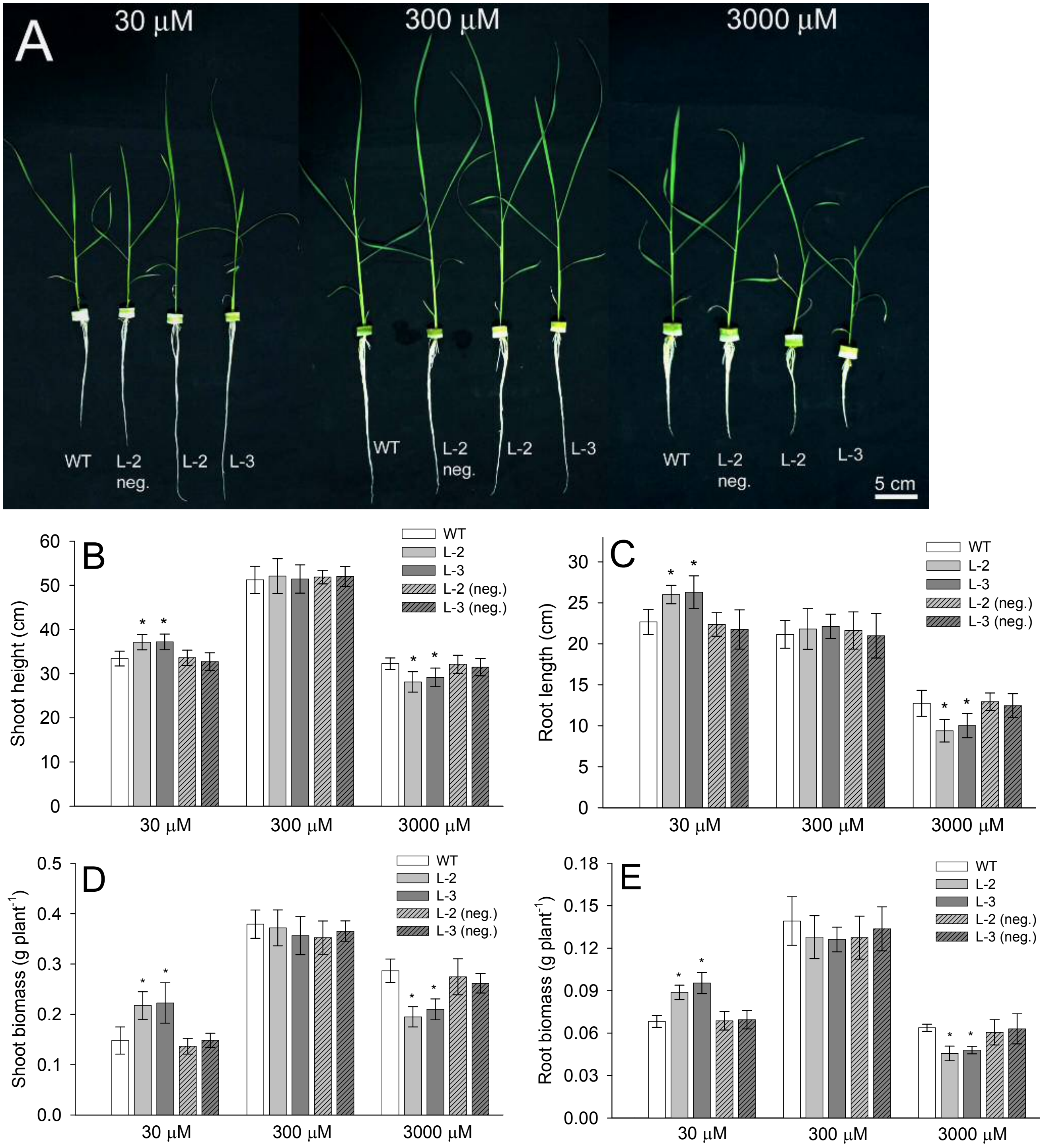

Supplementary Fig. 3

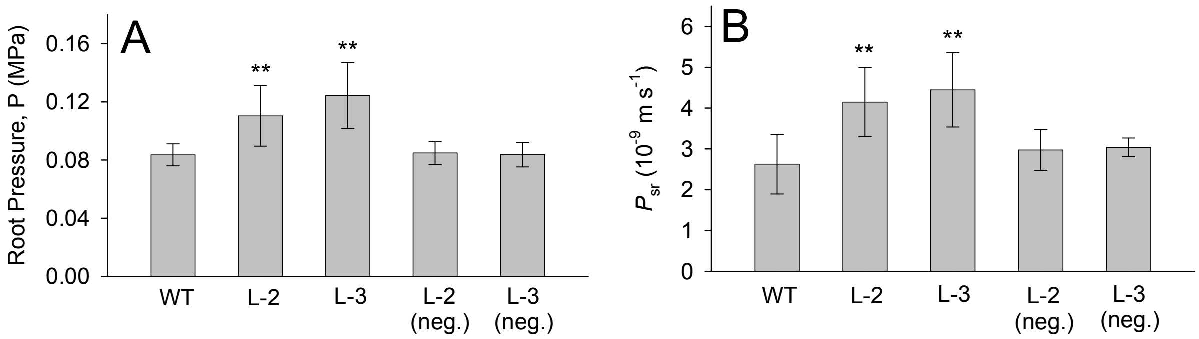

Supplementary Fig. 4

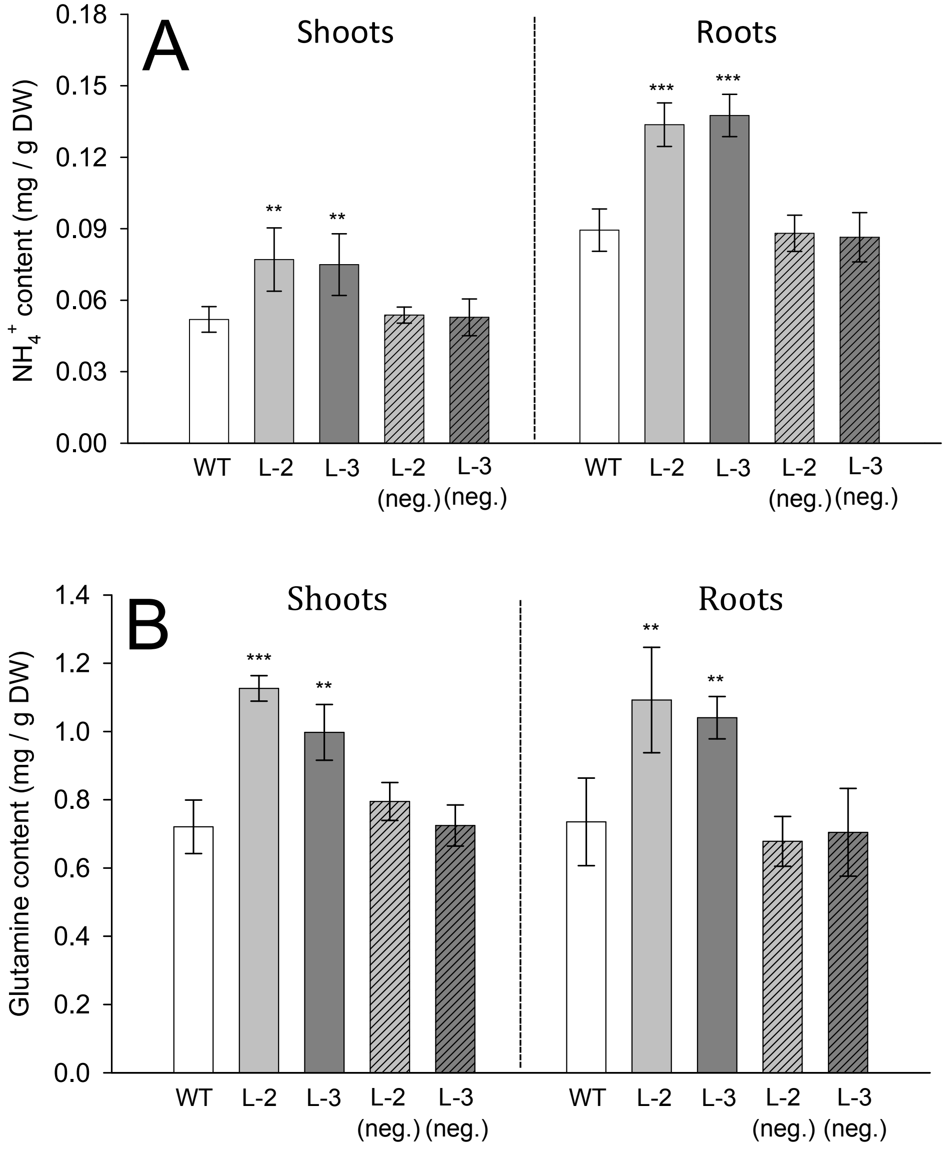

Supplementary Fig. 5

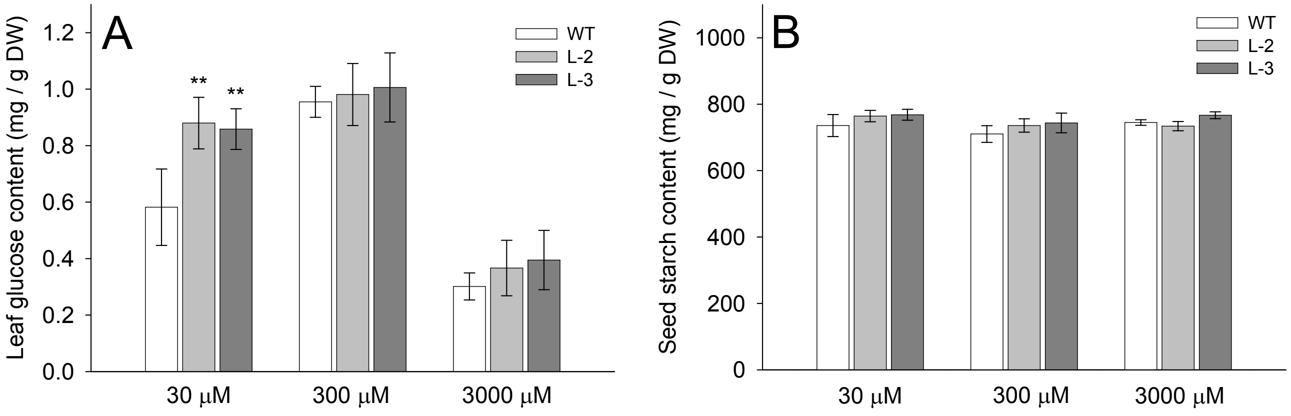

Supplementary Fig. 6

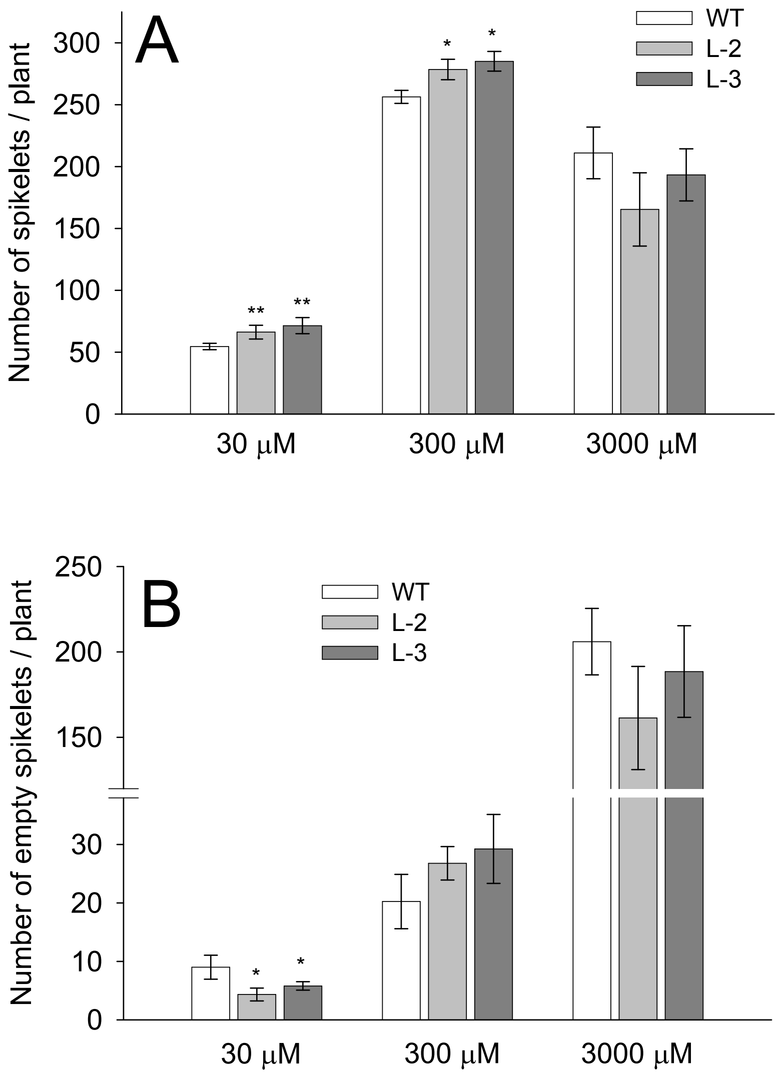

Supplementary Fig. 7
